# Warming and Drought Stress Modify Scent and Rewards in Flowers of Highbush Blueberry Affecting Pollinator Preferences

**DOI:** 10.3390/plants15111719

**Published:** 2026-06-02

**Authors:** Marcia González-Teuber, Felipe Torres Calisto, Camila Gálvez, Francisca Agüero-Hidalgo, María Victoria Gangas, Jan Bergmann

**Affiliations:** 1 Facultad de Ciencias Biológicas, Pontificia Universidad Católica de Chile, Santiago 8331150, Chile; felipetorres@uc.cl (F.T.C.); fagueroh@estudiante.uc.cl (F.A.-H.); mariavictoria.gangas@gmail.com (M.V.G.); 2 Instituto de Química, Facultad de Ciencias, Pontificia Universidad Católica de Valparaíso, Valparaíso 2340000, Chile; camila.galvez.j@mail.pucv.cl

**Keywords:** climate change, floral scent, pollen, nectar, pollinators, highbush blueberry

## Abstract

Changes in environmental temperatures and water availability can disrupt plant–pollinator interactions by altering floral attractive and rewarding traits. Here, we investigated the effects of warming and drought on floral scent and rewards in *Vaccinum corymbosum* (an entomophilous crop), and how these changes affect pollinator behavior. Plants were exposed to two temperatures (24 °C and 28 °C) and two watering treatments (optimal watering, W+, and water stress, W−). We measured floral volatiles, pollen and nectar quantity, as well as the nutritional composition of pollen (C, carbon, and N, nitrogen percentage) and nectar (hexose-to-amino acids ratio). Bioassays with honeybees were conducted to assess responses to the attractive and rewarding traits specific to each treatment. Floral volatiles significantly increased at 28° W+; nevertheless, they declined under the combination of both warming and drought. Pollen and nectar production were only negatively affected by warming. Pollen’s nutritional composition was negatively affected by the interaction of both stresses, with greater reductions in % C and N occurring when both stresses were combined. We observed that the synthetic floral scent representing the blend emitted by flowers under 28° W+ conditions, at low concentrations, attracted the highest percentage of honeybees. Additionally, honeybees tended to visit artificial diets of pollen with a more nutritious composition (50% carbon and 6% nitrogen), as found in 24° W+. We showed that changes in the composition of floral scent and pollen in varieties of *V. corymbosum* affected pollinator preferences in laboratory bioassays. This study contributes to our understanding of how climate change may impact trophic interactions by showing that changes in floral traits are associated with alterations in pollinator preferences.

## 1. Introduction

In the context of climate change, plants are experiencing increases in temperatures and water stress, which, in combination, might be more detrimental to plants than either stress alone [1]. The plant reproductive stage has been reported to be highly susceptible to abiotic stresses [2,3,4], potentially altering plant–insect interactions [5,6,7]. In terrestrial ecosystems, insect pollination is a crucial service for fruit crop production, which contributes to an estimated 30% of global crop production [8,9]. Furthermore, 75% of all crop species used for human consumption worldwide require insect pollination [10]. Therefore, for crops that rely on animal pollinators for fruit production, it is essential to understand how climate change may affect floral signals and rewards, and whether these changes may alter pollinator behavior.

Floral scent (=volatile organic compounds, VOCs) enhances the long-distance attraction of pollinators to flowers, whereas floral rewards, including nectar and pollen, provide nutritional resources for pollinators [11,12,13]. Variations in floral scent and rewards have often been reported in response to the individual effects of warming and drought [14,15,16,17]. Warming generally increases the total emission of floral volatiles up to a species-specific optimum temperature [18]. Above this optimum, floral emission declines [18]. For example, floral scent compounds emitted by *Petunia axillaris* flowers reached a maximum at 25 °C or 30 °C, but declined drastically at 35 °C [18]. Under drought conditions, the emission of floral scents appears to depend on the specific volatile compound involved. Some classes of compounds, such as the attractive terpenoids and benzenoids, decrease in response to water stress, whereas others, such as fatty acid derivatives, increase [15,16,19,20]. Like floral volatiles, nectar volume has been reported to increase with rising temperatures, but only up to an optimum temperature [21,22]. Under drought stress, nectar volume and its sugar composition, including hexoses and amino acids, often decrease [16,23,24]. Pollen quantity and viability are highly susceptible to warming and drought [17,25]. Nevertheless, the effects of these stresses on pollen nutritional composition—which is crucial for pollinator foraging—have not been extensively examined. Since pollinator preferences are often influenced by floral scent, as well as by the quantity and quality (i.e., nutritional composition) of floral rewards [24,26], variations in floral traits are likely to have important consequences for plant–pollinator interactions.

Plant–pollinator interactions can be reshaped by climate change [16,19,27], either increasing or decreasing pollinator attraction and preference for flowers. Several studies have reported that declines in nectar and pollen quantity in flowers subjected to warming and drought may explain reductions in pollinator visitation rates and preferences [16,24,25]. Climate change-induced shifts in floral scent and their cascade effects on pollinators have been far less investigated. An in situ long-term experiment showed that drought altered floral emissions in three Mediterranean plant species, which was likely associated with changes in pollinator behavior. Specifically, *Apis mellifera* and *Bombus terrestris* visited more flowers in control plots than in drought-treated plots [19].

*Vaccinium corymbosum* (the highbush commercial blueberry) is the most economically important fruit crop within the family Ericaceae. Highbush blueberry is an entomophilous species, i.e., it depends on insect pollination [28]. The primary pollinators in commercial plantations are bumblebees and honeybees [29]. The aim of this study is to understand the individual and combined effects of warming and drought stress during floral development in *Vaccinium corymbosum*. We measured floral volatile production, nectar and pollen quantities, and nutritional composition (hexose-to-amino acids ratio in nectar; carbon and nitrogen content in pollen). Additionally, laboratory bioassays were conducted to assess how changes in the composition of floral scent and rewards affect honeybees’ preferences. Our hypothesis is that when warming and drought stress act together, they are more harmful to flowers than when they act alone, resulting in reduced floral scent emission and rewards, which in turn decreases honeybees’ preferences. Specifically, we addressed the following questions: (1) Does the combination of warming and drought stress lead to a greater decrease in the emission of floral volatiles and the production of pollen and nectar compared to individual stress? (2) How is the chemical composition of floral scent and rewards affected by the combination of warming and drought stress? (3) Do changes in the composition of floral scent and rewards cause variations in honeybees’ preferences in laboratory bioassays?

## 2. Materials and Methods

### 2.1. Study Systems and Growing Conditions

*Vaccinium corymbosum* is a perennial shrub of the family Ericaceae, a native North American species with entomophilous flowers, which depend on pollinators for reproduction [28]. Blueberry flowering occurs in early spring [30], and flowers primarily exhibit protandry, whereby flowers release pollen before stigmas are receptive [31]. Insect visitation is essential for pollination of blueberries, since insects pick up, transport, and deposit pollen, leading to further pollen germination and fertilization that results in increased fruit set, size, and ripening of berries [32]. It has been reported that the optimal growth temperature ranges between 25 °C and 30 °C for *V. corymbosum*, and that it is highly sensitive to temperatures above 30 °C [33]. Plants of two different cultivars of *V. corymbosum* were used in this study (var. Cargo and var. Keecrisp for measurements of volatiles and rewards, respectively). The plants were approximately two years old, averaging 30 cm in height at the start of the experiment. Plants of both varieties were grown in 963.54 cm^3^ pots (base area: 56.25 cm^2^; opening area: 100 cm^2^; height: 12.5 cm), with a substrate composed of pine bark (weight ~300 g; pH 4.5–5.5). Before starting the experiments, all plants received homogeneous irrigation (80% field capacity). Because each plant produced only a small number of flowers (2–5 per individual), it was not possible to perform all measurements on the same plant within a single flowering season. Therefore, two controlled experiments were conducted in different years: the first (2024) measured floral rewards, and the second (2025) measured floral volatiles. Both experiments were developed under the same environmental conditions (see below). For the bioassays, adult workers of *Apis mellifera* (honeybees), from commercial hives (Colmenares Apiquirí, Lampa, Chile), were used. Prior to the assays, honeybees were acclimated in mesh cages (40 cm × 40 cm × 40 cm) with passive ventilation for 48 h and fed with a 20% sucrose solution (*p*/*v*) [34].

### 2.2. Experimental Design and Treatments

Plants during the floral transition (marked by floral bud emergence) were subjected to the individual and combined effects of warming and drought stress for ~14 days. Plants were grown under two different environmental temperatures: (1) 24 °C/20 °C day/night (=optimal temperature) and (2) 28 °C/24 °C day/night (high temperature). Aralab FitoClima Multi-Tier Plant Growth chambers were used in this study; they were designed for research requiring precise control of environmental conditions, including temperature, humidity, light intensity, and CO_2_. Both chambers had the same humidity (50%), light intensity (200 µmol m^−2^ s^−1^), and CO_2_ concentration (400 ppm). We selected 28 °C as a warming treatment because it is a high temperature within the optimal temperature range for *V. corymbosum*. Then, plants growing at both temperatures were either subjected to optimal watering (W+, 80% of field capacity) or to drought stress (W−, 40% of field capacity). Plants were randomly distributed within each chamber. The plants were irrigated twice a week, but with the aforementioned difference in the percentage of field capacity. Soil water content was quantified using a soil moisture meter (PMS710) since the start of the watering treatment. A reduction in the soil water content of 50% would constitute moderate drought stress for plants. In total, four plant treatments were obtained: 24° W+, 24° W−, 28° W+, and 28° W−. Each group started initially with 10 individual plants. Each group of plants experienced 100% survival within the 14 days of the experiment. Plants from certain groups produced fewer than three flowers, which limited the number of replicates for some measurements. All measurements (VOCs, pollen, and nectar) were made by pooling 3–5 flowers per plant.

### 2.3. Measurements of Floral Volatiles

Floral volatiles (ng h^−1^ g^−1^ dry weight) of *V. corymbosum* (var. Cargo) were collected from 3 to 5 flowers per individual plant (N = 3 plants per treatment, except for the 24° W+ treatment, where it was 2 due to logistical constraints related to the volatile sample procedure). Flowers from each treatment were cut (just before volatile collection) and placed in PET bags (Toppits^®^ Bratschlauch, Melitta, Minden, Germany), then secured at the top and bottom with a cable binder. Floral VOC emission was measured using a push–pull system. In both systems, charcoal-filtered air was continuously pumped into the bags (0.4 L/min flow rate). At the same time, air was pumped out through VOC traps (0.3 L/min flow rate), consisting of 25 mg of PoraPak-Q absorbent (Alltech, Deerfield, IL, USA) inserted into Teflon tubes. Floral volatiles were collected for 24 h. After VOC collection, the VOC traps were eluted with 200 µL dichloromethane containing *n*-octane as an internal standard (Sigma–Aldrich, Santiago, Chile, 5 ng µL^−1^). VOC extracts were analyzed using a gas chromatograph coupled to a mass spectrometer (GCMS-QP2010 Ultra, Shimadzu, Kyoto, Japan) equipped with an Rxi-5 ms column of 30 m × 0.25 mm × 2.65 μm (Restek, Bellefonte, PA, USA). The carrier gas was helium with a flow rate of 40 cm/s. The temperature program started at 50 °C, was maintained for 5 min, and then heated at 5 °C/min up to 150 °C. Then the temperature increased at 15 °C/min to 270 °C and was maintained for 10 min. The program duration was 43 min, operating in splitless mode. The mass spectra were obtained with an electron impact of 70 eV. The data obtained for a certain compound comprised the gas chromatographic retention index and the mass spectrum, which were compared to values found in the literature and in a mass spectral database (The National Institute of Standards and Technology (NIST11)). Tentative identifications were corroborated by comparison of the retention index and mass spectrum with an authentic reference compound whenever available. The approximate total amount of each compound in each sample was calculated by relating its peak area to that of the internal standard [35].

### 2.4. Measurement of Pollen and Nectar Quantity, and Chemical Composition

For pollen quantification, 3–6 flowers per plant of *V. corymbosum* (var. Keecrisp) were collected and pooled one day prior to anthesis. Pollen quantity was determined in 7 plants per treatment. The tips of the anthers were removed gently and placed in a 1.5 mL Eppendorf tube. Then, the tubes were incubated overnight at 28 °C to dry the anthers and promote their opening. They were then shaken at room temperature in a thermoshaker for 20 min at 600 rpm. Pollen was resuspended in 50 µL of 5% Tween 20; then centrifuged at 10,000 rpm for 10 min. A total of 2 µL of the resuspended pollen was placed in a Neubauer chamber, covered with a coverslip, and the total number of pollen grains was counted. The number of pollen grains was calculated using the known dimensions of the Neubauer chamber (0.00025 mm^3^ per quadrant) and adjusted by the dilution factor corresponding to the initial total suspension volume (50 µL). For pollen chemical composition, the percentage (%) of carbon (C) and nitrogen (N) was measured in pollen samples (1 mg per sample) (N = 7 plants per treatment) by dry combustion with a Perkin Elmer Elemental Analyzer (EA 2400 Series II CHNS/O Analyzer).

The collection and quantification of nectar were conducted as follows. Three days after anthesis, when *V. corymbosum* flowers produce the most nectar, 3–6 flowers were taken from each plant. Individual flowers typically remain open for 5–10 days, depending on the treatment; nevertheless, the highest nectar production, regardless of treatment, was approximately between days 3–4 (previous observation by our group). Nectar was quantified in 7 plants per treatment, except for the 28° W− treatment, where it was quantified in 5 plants due to fewer flowers produced by plants. Nectar production (mg g^−1^ floral dry weight) was determined as the amount of soluble solids per gram flower dry mass, by quantifying the nectar volume with microcapillaries and the nectar concentration with a refractometer (Atago Co., Ltd., Tokyo, Japan) as described previously [36]. Flowers that had produced the nectar were dried at 50 °C for 24 h and then weighed. Floral nectar per plant was determined by averaging the nectar production of 3–5 flowers. Floral nectar was stored at −20 °C for further chemical analysis. A total of 10 µL of nectar was used for the determination of total free amino acids (mg) by the ninhydrin colorimetric method, according to Sun et al. (2006) [37]. Absorbance was measured at 570 nm using an Infinite 200 PRO (Tecan). For analysis of total hexoses (mg), 10 µL of nectar was extracted with methanol/chloroform/water solution (12:5:3 *v*/*v*/*v*) [38,39] and determined using phenol 2% and sulfuric acid [38,39]. Absorbance was measured at 490 nm using an Infinite 200 PRO (Tecan) with sucrose as a standard. Then, the ratio of amino acids to hexoses (mg^−1^) was calculated.

### 2.5. Synthetic Scents and Olfactometry Assays

Simplified synthetic blends were used to test whether treatment-dependent differences in the relative composition of floral VOCs were sufficient to alter honeybee behavior, following the general rationale of floral scent manipulation studies [40]. Synthetic blends help to gain an understanding of the biological activity of relevant compounds identified in the chemical analysis. Four synthetic blends of different composition were created using seven compounds identified from the floral scent of *V. corymbosum* (α-pinene, sabinene, β-pinene, β-myrcene, (*E*)-β-ocimene, γ-terpinene, and (*E*)-β-caryophyllene) to represent the four plant treatments (24° W+, 24° W−, 28° W+, 28° W−), based on the different relative ratios of compounds found in the floral scent for each treatment (see Tables S1 and S2). These compounds were selected for the bioassay because they greatly differed among the four treatments (see Table S2). Furthermore, the selected VOCs represent common volatiles found in blueberry flowers [35,41]. Two different concentrations were used to prepare each blend corresponding to each plant treatment (low: 100 µg mL^−1^ and high: 1000 µg mL^−1^). Chemical standards for the seven compounds (Sigma–Aldrich Chile) were diluted in hexane and mixed according to the relative proportions for each treatment (Table S3). Behavioral assays were conducted using a four-arm olfactometer; the airflow-driven odor delivery was consistent with four-arm olfactometer assays previously used for honeybees [42]. Recommendations for insect olfactometer bioassays, including airflow equalization, treatment randomization, and cleaning between trials [43], were applied. For each choice, we offered 10 µL of each synthetic scent on filter paper. In each trial, a single honeybee was released at the center of the arena and allowed to move freely for 180 s. Each honeybee was used only once to ensure independent trials. During each trial, the choice made by honeybees was recorded. A choice was considered when the honeybee remained in the first arm selected for at least 15 s. If the honeybee did not select any arm, it was considered as no choice. A total of 50 individual honeybees were tested across all comparisons. After each trial, filter papers were replaced, and 10 µL of the corresponding synthetic scent was applied again. To avoid positional bias, the positions of the treatments were randomized within replicates.

### 2.6. Cafeteria Style Experiments

Behavioral responses of honeybees to variations in floral rewards were assessed using ‘cafeteria’-style experiments under laboratory conditions [44]. Three artificial pollen diets were created, with the aim of imitating the results obtained by the elemental analysis. The diets were prepared by dissolving sucrose and a mixture of 10 amino acids in agar to achieve a texture similar to natural pollen [45,46]. Agar is widely used in artificial insect diets primarily as a gelling agent. While it is a polysaccharide, it is generally considered a non-nutritive, inert component in insect diets, providing no significant carbon or nitrogen for growth [47]. Sucrose (Sigma–Aldrich Chile) was used as the C source, whereas 10 different amino acids (lysine, histidine, leucine, methionine, arginine, valine, threonine, isoleucine, phenylalanine, and tryptophane; Sigma–Aldrich Chile) were used as the N source. Three pollen diets were created to have similar relative proportions of carbon (C) and nitrogen (N) as the proportions found in pollen of *V. corymbosum* (see results below). The diets were the following: (1) Diet 1 with 30% C and 3% N, (2) Diet 2 with 40% C and 3% N, and (3) Diet 3 with 50% C and 6% N. Percentage composition analysis of sucrose and amino acids was used to calculate necessary % C and % N to simulate nutrient ratios (C: N ratio) found in pollen samples. For diet preparation, agar, sucrose, and amino acids (the compositions and quantities of all diet components are provided in Table S4) were weighed, thoroughly mixed and homogenized, and maintained as a powder. After preparation, diets were stored under refrigeration until use. Before feeding, refrigerated diets were conditioned to room temperature for 2 to 3 h. Then, the four diets were simultaneously offered to honeybees. For this, three butterfly flight cages (40 cm × 40 cm × 40 cm) were used as replicates, and the diets were placed equidistantly, each containing 5 mg of artificial pollen. Three different honeybee colonies (~95–155 honeybees per colony), with a previous fasting period of one hour, were released into each cage. Preferences of honeybees for each diet were recorded with HD cameras for t six consecutive hours (9:00 AM–15:00 PM), and the position of diets was rotated randomly across time [48,49]. The visitation rate (n° of visits h^−1^) was estimated as the number of honeybees that made physical contact with the diet for at least 3 s. Pollen consumption (ng bee^−1^ h^−1^) was determined as the diet mass consumed, calculated as the difference between the initial and final weight of each diet. Since the chemical composition of nectar (amino acid-to-hexose ratio) did not significantly differ among floral nectar coming from the four treatments (see below), no ‘cafeteria’-style experiment was performed for this reward.

### 2.7. Statistical Analysis

A two-way ANOVA was used to evaluate the effects of warming, drought stress, and their interactions on pollen quantity, nectar production, and pollen and nectar chemical composition, followed by Tukey’s HSD post hoc test. For volatile emissions, a Student’s *t*-test was used to compare differences between W+ and W− at each temperature. To analyze the effects of different artificial pollen diets on estimated visitation rates of honeybees, we used a GLMM (*glmmTMb*) using a Tweedie distribution and a log link function, followed by a Tukey’s HSD *post hoc* test. Effects of different artificial pollen diets on consumption rates by honeybees were analyzed with a GLMM (*glmmTMb*) with a gamma distribution and log link, followed by a Tukey’s HSD *post hoc* test. Pollinator preferences for different synthetic scents were analyzed using a Chi-square test. All analyses were performed using the R statistical program [50].

## 3. Results

### 3.1. Production of Total Scent Emission and Floral Rewards

**Figure 1:**
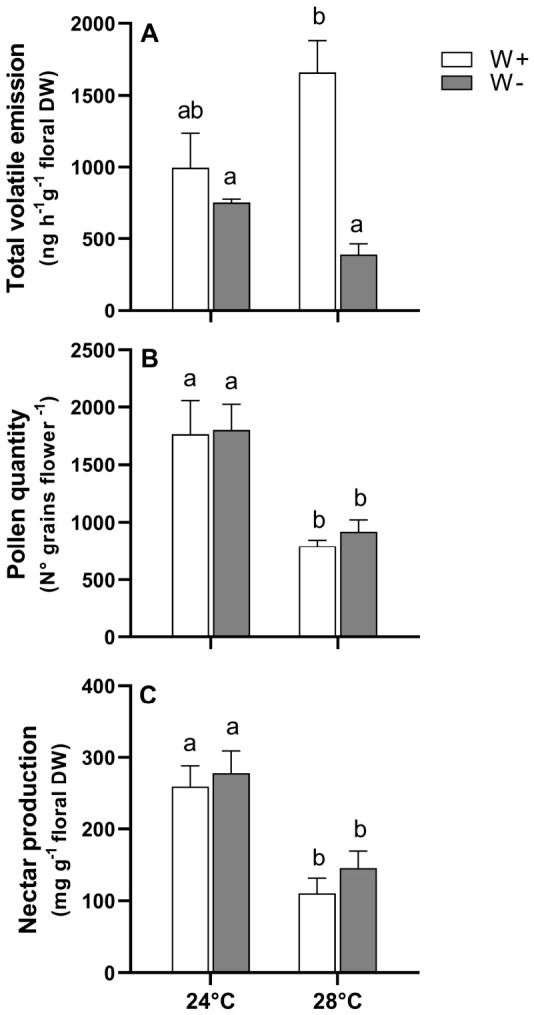
Effects of temperature (24°, 28 °C) and drought stress (optimal watering: W+, water stress: W−) on quantity of floral scent and rewards in plants of *Vaccinum corymbosum*: (**A**) total volatile emission, (**B**) pollen quantity, and (**C**) nectar production. Different letters indicate significant differences among treatments (*post hoc* Tukey’s HSD test after two-way ANOVA).

Total VOCs were not significantly affected by warming (F_1,7_ = 5.15, *p* = 0.057). Nevertheless, they were significantly affected by drought (F_1,7_ = 15.19, *p* = 0.005) and by the W × D interaction (F_1,7_ = 7.30, *p* = 0.030) (Figure 1A). Both pollen quantity and nectar production were significantly affected by warming (pollen: F_1,24_ = 23.40, *p* = 0.000; nectar: F_1,22_ = 24.40, *p* = 0.000), decreasing their quantity when plants were exposed to 28 °C (Figure 1B,C). Neither floral rewards were affected by drought (pollen: F_1,24_ = 0.172, *p* = 0.681; nectar: F_1,22_ = 1.996, *p* = 0.171) nor by the interaction W × D (pollen: F_1,24_ = 0.055, *p* = 0.816; nectar: F_1,22_ = 0.443, *p* = 0.512) (Figure 1B,C).

### 3.2. Composition of Floral Volatiles

**Figure 2:**
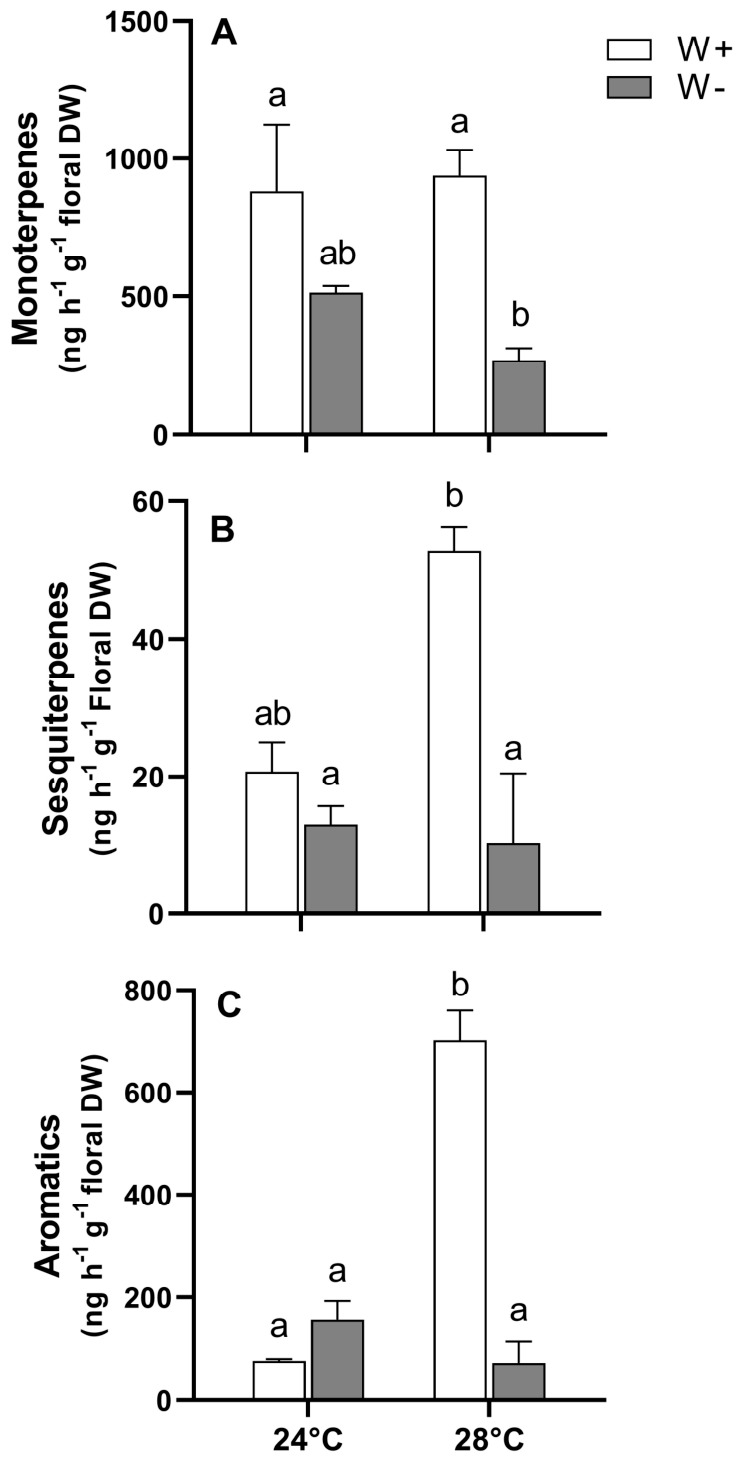
Effects of temperature (24 °C, 28 °C) and drought stress (optimal watering: W+, water stress: W−) on the composition of floral scent in plants of *Vaccinum corymbosum*: (**A**) monoterpenes, (**B**) sesquiterpenes and (**C**) aromatic compounds. Different letters indicate significant differences among treatments (*post hoc* Tukey’s HSD test after two-way ANOVA).

A total of 28 volatile compounds were identified from the flowers of *V. corymbosum*, which were classified by type of compound based on their presumed biosynthesis (Table S1). Among them, monoterpenes were the most abundant compounds, followed by aromatic compounds, sesquiterpenes, and fatty acid derivatives. Whereas only monoterpenes were significantly affected by drought (F_1,7_ = 26.8, *p* = 0.001), sesquiterpenes were significantly affected by both drought (F_1,7_ = 15.19, *p* = 0.005) and the W × D interaction (F_1,7_ = 7.30, *p* = 0.030) (Figure 2A,B). Neither warming (F_1,7_ = 0.86, *p* = 0.384) nor the interaction W × D (F_1,7_ = 2.27, *p* = 0.174) significantly affected monoterpenes. Warming did not have a significant effect on sesquiterpenes (F_1,7_ = 5.15, *p* = 0.057). Aromatic compounds, by contrast, were significantly affected by warming (F_1,7_ = 35.09, *p* = 0.000), drought (F_1,7_ = 36.25, *p* = 0.000), and by the W × D interaction (F_1,7_ = 60.15, *p* = 0.000) (Figure 2C). For fatty acid derivatives, even when their emission showed different tendencies between treatments (24° W+: 18.50 ± 1.32 ng h^−1^ g^−1^ dry weight; 24° W−: 72.41 ± 9.73 ng h^−1^ g^−1^ dry weight; 28° W+: 51.06 ± 9.53 ng h^−1^ g^−1^ dry weight; 28° W− 37.65 ± 36.79 ng h^−1^ g^−1^ dry weight), no significant effects were observed for warming (F_1,7_ = 0.002, *p* = 0.961), drought (F_1,7_ = 0.828, *p* = 0.393), or for the interaction W × D (F_1,7_ = 2.288, *p* = 0.174). For individual compounds, significant differences in their emission are shown in Table S2.

### 3.3. Chemical Composition of Pollen and Nectar

**Figure 3:**
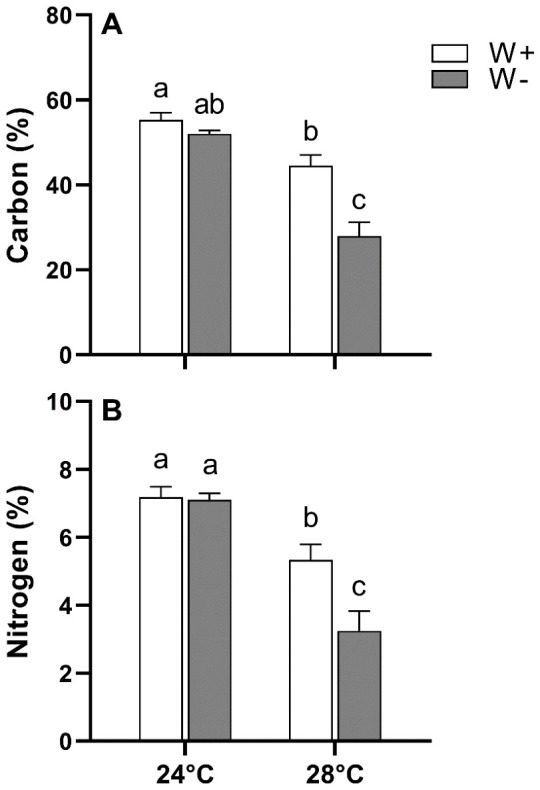
Effects of temperature (24 °C, 28 °C) and drought stress (optimal watering: W+, water stress: W−) on the nutritional composition of pollen in flowers of *Vaccinum corymbosum*: (**A**) carbon percentage and (**B**) nitrogen percentage. Error bar labels with different letters indicate significant differences among treatments (*post hoc* Tukey’s HSD test after two-way ANOVA).

Carbon (C) and nitrogen (N) contents in pollen were significantly affected by warming (C: F_1,24_ = 58.49, *p* = 0.000; N: F_1,24_ = 47.53, *p* = 0.000) drought (C: F_1,24_ = 18.80, *p* = 0.000; N: F_1,24_ = 6.875, *p* = 0.014) and the interaction of W × D (C: F_1,24_ = 8.34; *p* = 0.008; N: F_1,24_ = 5.998, *p* = 0.021). At 24 °C, there was no significant difference in the C and N content between W+ and W−, but at 28 °C, both C and N significantly decreased, with even lower levels at 28° W− than at 28° W+ (Figure 3A,B). The amino acid-to-hexose ratio in nectar did not differ among for four treatments (24° W+: 0.014 ± 0.005; 24° W−: 0.0082 ± 0.004; 28° W+: 0.0080 ± 0.002; 28° W−: 0.0054 ± 0.03), with no significant effect of warming (F_1,18_ = 0.914, *p* = 0.351), drought (F_1,18_ = 0.820, *p* = 0.377), nor the interaction W × D (F_1,18_ = 0.116, *p* = 0.736).

### 3.4. Attraction of Pollinators to VOCs and Rewards

**Figure 4:**
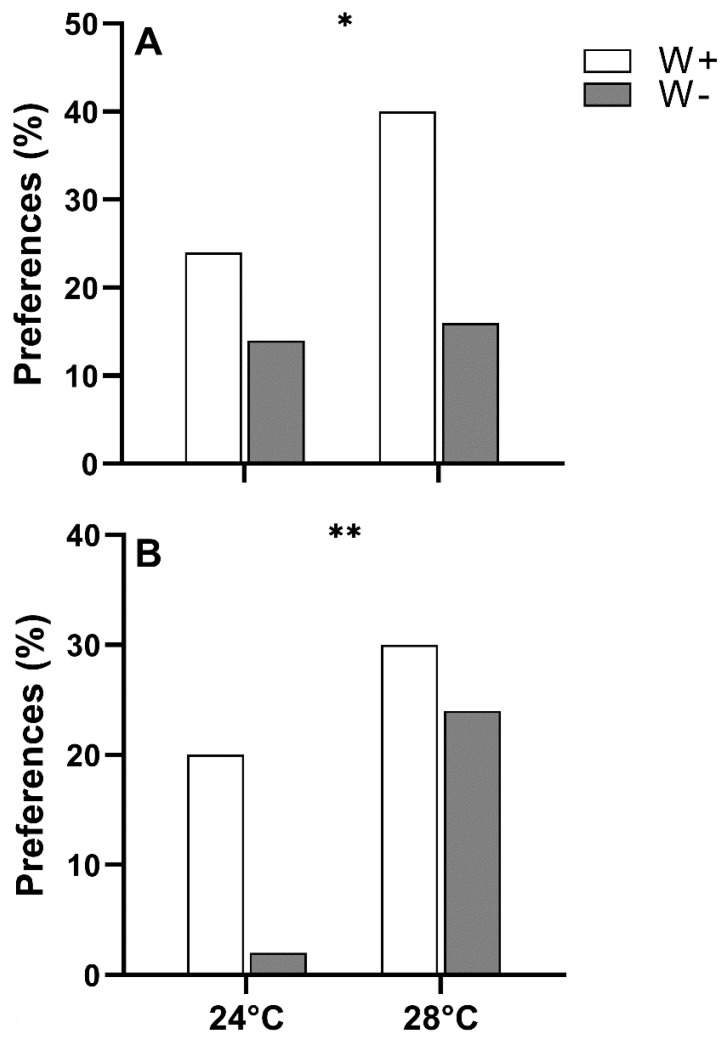
Effects of temperature (24 °C, 28 °C) and drought stress (optimal watering: W+, water stress: W−) on pollinator (=honeybees) preferences to four synthetic blends representing the four plant treatments (24° W+, 24° W−, 28° W+, 28° W−), based on the different relative ratios of compounds found in floral scent for each treatment (Table S2): (**A**) synthetic blends at low concentration (100 µg mL^−1^) and (**B**) synthetic blends at high concentration (1000 µg mL^−1^). Asterisks indicate the significance level of differences among four synthetic blends: ** *p* < 0.01, * *p* < 0.05 (Chi-square tests).

**Figure 5:**
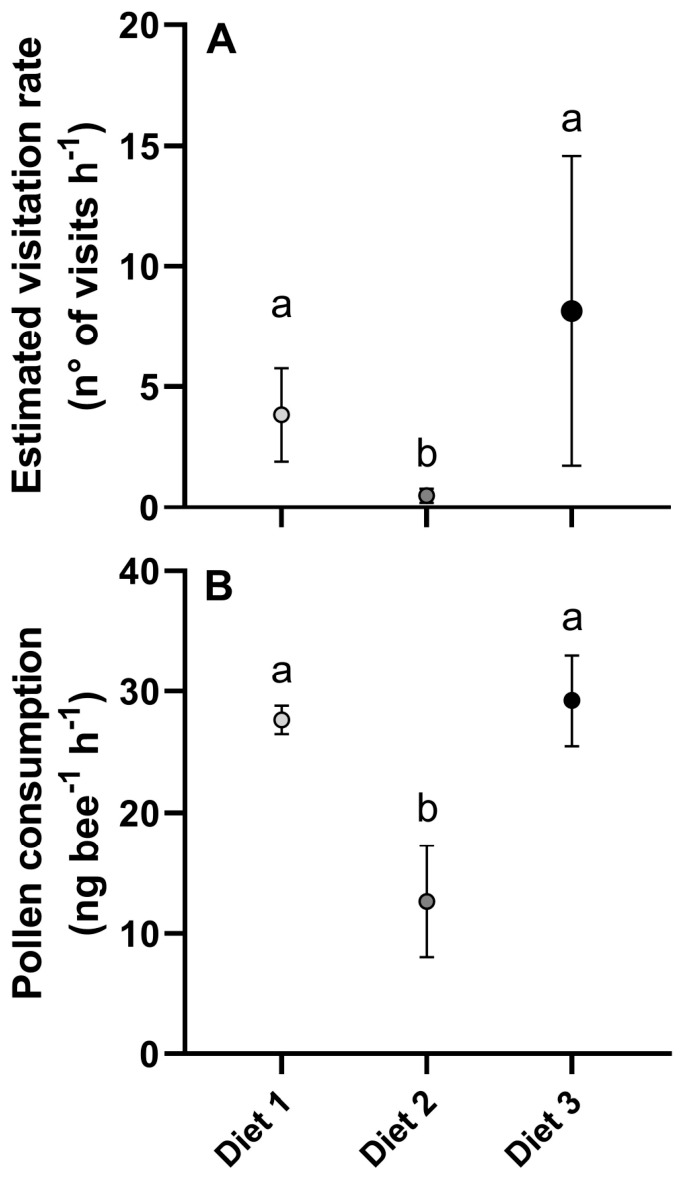
Effects of temperature (24 °C, 28 °C) and drought stress (optimal watering: W+, water stress: W−) on the (**A**) estimated visitation rate and (**B**) consumption rate of pollinators (=honeybees) to different pollen artificial diets. Diets 1–3 were based on agar but had similar relative proportions of carbon and nitrogen % as the proportions found in pollen of *V. corymbosum* (see Figure 3). Diet 1 with 30% C and 3% N, diet 2 with 40% C and 3% N, and diet 3 with 50% C and 6% N. Error bar labels with different letters indicate significant differences among treatments (*post hoc* Tukey’s HSD test after a GLMM).

For olfactometry assays, when synthetic blends were offered at a low concentration, honeybees showed significantly higher preferences for the blend imitating 28° W+ (Chi^2^ = 9.489, df = 3, *p* = 0.0234) than for other blends (Figure 4A). Contrary, when synthetic blends were offered at a high concentration, honeybees showed similar preferences for blends imitating 24° W+, 28° W+, and 28° W− (Chi^2^ = 11.47, df = 3, *p* = 0.009), with a significant decrease in their preferences for the 24° W- treatment (Figure 4B). Regarding artificial pollen diets, the visitation rate by honeybees was significantly different among diets (Chi^2^ = 17.57, df = 3, *p* < 0.001), with a clear tendency for diet 3 (50% C 6% N) to be more visited than the control and diet 2 (Figure 5A). Similarly, the pollen consumption rate also differed among diets (Chi^2^ = 19.93, df = 3, *p* < 0.001), with diets 1 (30% C 3% N) and 3 (50% C 6% N) being significantly preferred over the control and diet 2 (40% C 3% N) (Figure 5B).

## 4. Discussion

### 4.1. Floral Scent and Attraction to Pollinators

Besides visual traits, floral scent is a key advertisement trait for pollinators [51]. Nevertheless, floral scent can change in response to environmental factors, potentially impacting plant–pollinator interactions [52,53]. In this study, we showed that increasing temperature (28 °C) without drought stress led to a significant increase in total floral scent emission in highbush blueberry plants, nearly twice that of plants grown at the optimal temperature (24 °C) without drought stress. In contrast, the combination of warming and drought led to a significant decrease in the production of volatile compounds. This pattern, i.e., an increase under warming-only conditions and a decrease under combined warming–drought stress, was observed across almost all compound classes, including monoterpenes, sesquiterpenes, and aromatic compounds. Other studies are consistent with our findings, showing that higher temperatures increase floral scent emission [54,55]. This response is likely related to enhanced volatilization and diffusion of compounds at higher temperatures. In addition, volatile emission is regulated by biosynthetic pathways that are temperature-dependent, as enzyme activity generally increases with temperature [56]. However, beyond an optimum temperature, enzyme activity declines, potentially leading to reduced volatile emissions. Drought stress triggers complex physiological and molecular alterations in plants, which likely include the downregulation of scent-related genes, as metabolic resources are shifted from specialized secondary metabolism toward stress-adaptation mechanisms [57]. For example, total terpenoid emission in the Mediterranean species *Quercus suber* and *Cistus ladanifer* decreased significantly under drought stress, although individual compounds were affected differently [58]. The combination of drought and a second abiotic stress, in this case, warming, may aggravate these responses, resulting in greater reductions in the total emission of attractive floral volatiles. Our study contributes to the understanding of the combined effects of multiple stress factors on floral volatile emissions. While studies investigating these effects are still scarce, available evidence—including this study—suggests that floral volatile profiles can be altered by the combination of different stresses (e.g., leaf and flower herbivory, drought, and leaf herbivory, or the interaction of climate change-related factors) [52,59].

Climate change-induced shifts in floral scent often reduce pollinator attraction and behavior [19]. For example, under natural conditions, plants of *Brassica napa* subjected to warming conditions (26 °C vs. 21 °C) emitted a different volatile blend with overall lower volatile emission and received fewer visits from bumblebees and butterflies [20]. In our laboratory bioassays, we observed that at the 10 µg dose, the synthetic floral scent mimicking the 28° W+ treatment attracted the highest proportion of *Apis mellifera* (40%) compared with the other treatments. Because the total amount of scent was kept constant, this result indicates differences in preferences for blend composition rather than dose. This effect may be partly explained by the presence and higher proportion of (*E*)-β-ocimene in this blend, a compound that was absent when flowers were subjected to drought stress. Ocimene is a common monoterpene emitted by a wide range of plant species across many families. There is substantial evidence for its role in the attraction of pollinators and as part of herbivore-induced plant indirect defense [60,61]. For example, flowers of *Bidens pilosa* var. *radiata* emitted high levels of (*E*)- and (*Z*)-β-ocimene, and both compounds act as key attractants for *Apis cerana,* highlighting their contribution to pollinator attraction [62]. Consistent with these results, when high concentrations of synthetic blueberry scents were presented to *Apis mellifera*, a clear avoidance was observed for the 24° W-blend, which received only 2% of the choices and was characterized by the absence of (*E*)-β-ocimene and a low concentration of the monoterpene γ-terpinene. Terpinene is also a key volatile component contributing to many floral scents [63,64], and was found at higher proportions for the 28° W-blend than in the 24° W-blend. Thus, based on changes in component ratios, our results suggest that (*E*)-β-ocimene and γ-terpinene are likely involved as olfactory signals in attracting specific pollinators in *Vaccinum corymbosum*. Nevertheless, due to the lack of GC–EAD screening, these conclusions are limited by the use of a predetermined synthetic blend. We cannot exclude the attractiveness of the other 21 volatile compounds that were not tested in our bioassays, and further research is required.

### 4.2. Floral Rewards and Pollinator Preferences

Negative effects of warming and drought stress on nectar and pollen quantity have been previously demonstrated [24,65]. While the sensitivity of pollen quantity, germination, and viability to warming is relatively well established in the literature [17,24,66], the effects of warming and drought on pollen nutritional composition, as well as on pollinator behavior, remain less studied. Nectar production, on the other hand, appears to be sensitive to both warming and drought [24,55,67]. For *V. corymbosum*, we observed that both pollen quantity and nectar production declined under warming (28 °C), with no negative effects of drought stress alone. The latter does not support our hypothesis that the combined stresses are more harmful to floral rewards than when they act individually. In *V. corymbosum*, the lack of significant effects of drought on pollen and nectar production may be due to the relatively moderate intensity of the drought treatment. The severity and duration of drought stress play a crucial role in determining changes in plant carbohydrate allocation and their distribution to different organs [68]. Descamps et al. (2021a) reported in *Borago officinalis* that increased temperature, but not water stress, induced a 50% decrease in pollen weight per flower [24]. Nectar volume, however, decreased under both warming and drought. Variations in nectar quantity may influence pollinator behavior, including in blueberry [69]. Lower preferences by pollinators have been linked to low nectar rewards, primarily because the cost of foraging (time and energy) exceeds the reward [70]. In nectar, we did not find any change in the amino acid-to-hexose ratio. In *Borago officinalis*, warming and drought resulted in a 60% decrease in total nectar sugar quantity and an increase in total amino acid concentration [24]. For pollen, while a high quantity can increase the likelihood of pollinator visits, some studies have shown that honeybees often select pollen based on its nutritional value [71]. Regarding the nutritional composition of pollen and nectar, we found that pollen was affected by warming and by the interaction between the two stressors. Warming alone caused a decrease in C% and N% in pollen, but the combination with drought stress further intensified this effect, with reductions of approximately 35% and 45% for carbon and nitrogen, respectively. For *V. corymbosum*, a previous study indicated that extreme warming (37.5 °C) led to lower concentrations of proteins, total amino acids, and several individual amino acids in pollen [72].

Pollinator behavior may be sensitive to shifts in the availability and nutritional quality of floral rewards [73]. Changes in nectar volume have been observed to affect flower visitation by insect pollinators and birds [73,74,75], while alterations in pollen quantity or nutritional composition can impact foraging efficiency and pollinator preferences [76]. A reduction in pollen per flower may increase the cost of visitation for pollinators by decreasing net energy gain per visit, as pollinators spend more time and energy searching for insufficient rewards [77]. Our laboratory bioassays showed that *Apis mellifera* tended to prefer artificial pollen diets with a more nutritious composition of carbohydrates and amino acids. Honeybee preferences for this diet may be strongly stimulated and conditioned by sugar concentration [78,79,80]. Bees use specialized receptors to evaluate sugar concentration and type in the diet, typically preferring high-concentration sources to maximize energy intake [80,81]. Changes in the nutritional composition of pollen could potentially affect pollinator attraction to stressed flowers under field conditions, although this remains to be tested.

## 5. Conclusions

Climate change will alter the way plants interact with higher trophic levels, such as herbivores and pollinators [20,82]. Here, we observed that climate change-related shifts in floral scent and rewards in both varieties of *Vaccinum corymbosum* affected honeybee preferences. Genetic differences between varieties, as well as year-to-year variation in growing conditions, may influence how varieties respond to stress; therefore, caution is needed when generalizing stress effects across the species. For floral volatiles and pollen nutritional composition, the combination of both stressors was more detrimental than either stress alone, and this was associated with changes in pollinator behavior in laboratory bioassays. In our study, pollinator visitation in planta was not assessed, and the relationship between our laboratory results and field conditions remains to be examined. For entomophilous plant species such as *V. corymbosum*, potential disruption of plant–pollinator interactions may be critical, as they rely on insect pollinators for reproduction. Therefore, further research on this crop is still needed to examine the direct and indirect effects of climate change on pollinators, as their decline could disrupt plant reproduction, reduce agricultural production, and threaten food security, biodiversity, and ecosystem stability.

## Data Availability

The raw data supporting the conclusions of this article will be made available by the authors on request.

## Supplementary Materials

The following supporting information can be downloaded at: https://www.mdpi.com/article/10.3390/plants15111719/s1, Table S1: Volatile organic compounds identified in floral samples from *Vaccinium corymbosum*; Table S2: Emission rate of VOCs (ng h^−1^ g^−1^ dry weight of flower) from flowers of *Vaccinum corymbosum* subjected to different stress treatments; Table S3: Percentage (%) of the synthetic scent created for each plant treatment; Table S4: Percentage (%) of agar, sucrose and amino acids used to create pollen artificial diets according to the pollen’s nutritional composition found for each plant treatment.
