# Supplementary material for: Warming and Drought Stress Modify Scent and Rewards in Flowers of Highbush Blueberry Affecting Pollinator Preferences
Source: Plants (Basel). 2026 Jun 2;15(11):1719. doi: 10.3390/plants15111719 (PMC13259225; doi:10.3390/plants15111719)
Supplement: Supplementary file 1 [file plants-15-01719-s001.zip › plants-4255187-supplementary.pdf]

Table S1: Volatile organic compounds identified in floral samples from *Vaccinium corymbosum*. (A) represents the tentative identification based on comparison of the retention index and mass spectrum with the NIST database, while (B) represents the compounds identified using pure standards of the compounds.

N°	RT (min)	Retention index	VOC	VOC family
1	8,77	927	α-Thujene ^{A}	Monoterpene
2	9,00	935	α-Pinene ^{B}	Monoterpene
3	9,53	948	Camphene ^{A}	Monoterpene
4	10,43	973	Sabinene ^{B}	Monoterpene
5	10,51	975	β-Pinene ^{B}	Monoterpene
6	11,08	991	β-Myrcene ^{B}	Monoterpene
7	11,65	1008	(*Z*)-3-Hexenyl acetate ^{B}	Fatty acid derived
8	11,95	1017	(*E*)-2-Hexenyl acetate ^{A}	Fatty acid derived
9	12,18	1023	*p*-Cymene ^{A}	Monoterpene
10	12,35	1028	Limonene ^{B}	Monoterpene
11	12,42	1030	Eucalyptol ^{B}	Monoterpene
12	13,04	1049	(*E*)-β-Ocimene ^{B}	Monoterpene
13	13,39	1059	γ-Terpinene ^{B}	Monoterpene
14	14,38	1088	α-Terpinolene ^{B}	Monoterpene
15	14,40	1089	p-Cymenene ^{A}	Aromatic
16	15,96	1138	Benzyl cyanide ^{B}	Aromatic
17	16,17	1145	Camphor ^{A}	Monoterpene
18	17,42	1185	p-Cymen-8-ol ^{A}	Monoterpene
19	19,74	1264	3-Ethylacetophenone ^{B}	Aromatic
20	19,93	1271	Cinnamaldehyde ^{A}	Aromatic
21	20,26	1282	4-Ethylacetophenone ^{B}	Aromatic
22	20,90	1305	Cinnamyl alcohol ^{A}	Aromatic
23	22,69	1371	3-Phenylpropyl acetate ^{A}	Aromatic
24	24,09	1426	(*E*)-β-Caryophyllene ^{B}	Sesquiterpene
25	24,62	1448	Cinnamyl acetate ^{A}	Aromatic
26	24,97	1463	α-Caryophyllene ^{B}	Sesquiterpene
27	25,61	1489	Germacrene D ^{A}	Sesquiterpene
28	26,06	1512	α-Farnesene ^{B}	Sesquiterpene

Table S2: Emission rate of VOCs (ng h-1 g-1 dry weight of flower) from flowers of *Vaccinum corymbosum* subjected to different stress treatments. VOC concentrations are reported as the mean ± SE. Asterisks indicate the significance level of differences for each factor (W: warming, D: drought, W x D: interaction): ***p < 0.001, **p < 0.01, *p < 0.05, ns = no significant difference (after a two-way ANOVA).

VOC	24°W-	24°W+	28°W-	28°W+	p-value
α-Thujene	3,1 ± 0,57	12,0 ± 2,16	8,72 ± 0,89	11,1 ± 3,82	ns
α-Pinene	18,1 ± 5,19	9,4 ± 5,45	3,76 ± 0,30	5,6 ± 3,25	**W***
Camphene	8,2 ± 2,15	7,5 ± 4,35	53,8 ± 38,10	0,5 ± 0,5	ns
Sabinene	30,2 ± 2,35	18,6 ± 10,75	16,8 ± 6,86	61,57 ± 9,64	**D*****
β-Pinene	24,5 ± 14,18	0	0	1,1 ± 0,78	ns
β-Myrcene	328,3 ± 46,01	673,2 ± 128,0	138,2 ± 2,84	609,8 ± 80,41	**D****
(*Z*)-3-Hexenyl acetate	53,5 ± 4,83	18,5 ± 0,76	30,9 ± 21,27	41,6 ± 7,42	ns
(*E*)-2-Hexenyl acetate	18,9 ± 3,48	0	6,7 ± 4,74	10,4 ± 3,91	ns
p-Cymene	9,7 ± 0,90	4,9 ± 2,85	9,8 ± 3,09	12,7 ± 2,82	ns
Limonene	44,1 ± 8,12	65,3 ± 8,73	54,5 ± 4,03	65,9 ± 5,22	ns
Eucalyptol	17,1 ± 12,12	0	0	0,3 ± 0,36	ns
(*E*)-β-Ocimene	0	13,0 ± 1,29	0	26,6 ± 2,26	**W**<br>D***<br>WxD****
γ-Terpinene	16,7 ± 1,33	37,0 ± 2,43	14,3 ± 10,13	40,6 ± 5,91	**D***
α-Terpinolene	17,2 ± 4,07	49,2 ± 0,42	31,4 ± 16,59	65,2 ± 3,50	**D***
p-Cymenene	10,4 ± 0,76	5,1 ± 3,50	9,9 ± 6,07	28,4 ± 2,47	ns
Benzyl cyanide	4,4 ± 0,91	5,5 ± 1,15	2,7 ± 1,94	5,4 ± 0,84	ns
Camphor	10,3 ± 7,30	0	0	0	-
p-Cymene-8-ol	2,1 ± 0,14	1,4 ± 0,82	2,8 ± 1,99	8,5 ± 0,83	ns
3-Ethylacetophenone	15,6 ± 3,98	14,1 ± 0,53	22,1 ± 6,20	22,8 ± 2,42	ns
Cinnamaldehyde	31,3 ± 8,22	1,4 ± 1,48	13,8 ± 13,83	51,7 ± 9,73	**WxD***
4-Ethylacetophenone	16,8 ± 5,86	22,9 ± 3,96	20,9 ± 6,59	28,5 ± 3,38	ns
Cinnamyl alcohol	108,5 ± 25,90	18,5 ± 10,71	0	497,1 ± 55,32	**W**<br>D***<br>WxD*****
3-Phenylpropyl acetate	2,0 ± 0,11	7,1 ± 4,11	1,7 ± 1,23	8,4 ± 2,39	ns
(*E*)-β-Caryophyllene	7,2 ± 1,51	10,2 ± 2,20	5,4 ± 3,84	17,7 ± 7,78	ns
Cinnamyl acetate	4,5 ± 1,97	0	0	61,9 ± 9,57	**W**<br>D****

					**WxD***
α-Caryophyllene	1,6 ± 0,56	1,1 ± 0,19	0,6 ± 0,46	5,5 ± 1	ns
Germacrene D	3,3 ± 0,53	5,4 ± 0,51	2,9 ± 2,05	24,8 ± 3,69	**W*<br>D**<br>WxD***
α-Farnesene	3,9 ± 0,60	3,9 ± 0,05	1,2 ± 0,85	5,438 ± 0,88	ns

Table S3: Percentage (%) of the synthetic scent created for each plant treatment. Synthetic scents were created with those compounds whose emission were significantly different among treatments (Table S2) or showed very contrasting emissions (Table S2). All compounds used were obtained from Sigma Aldrich.

VOC	24°W+	24°W-	28°W+	28°W-
α-Pinene	2,39	4,25	0,75	2,11
Sabinene	4,72	7,10	8,07	9,41
β-Pinene	0	5,78	0,13	0
β-Myrcene	85,25	77,27	79,9	77,41
(*E*)-β-Ocimene	1,66	0	3,49	0
γ-Terpinene	4,69	3,93	5,33	8,02
(*E*)-β-Caryophyllene	1,29	1,70	2,33	3,05
Total	100%	100%	100%	100%

Table S4: Percentage (%) of agar, sucrose and amino acids used to create pollen artificial diets according to the pollen's nutritional composition found for each plant treatment (see Figure 3). Sucrose determines the final % of carbon (C) in the artificial diet, whereas amino acids contribute to both carbon (C) and nitrogen (N) content. Agar, by contrast, is an inert, non-nutritive component that does not supply usable carbon or nitrogen to insects. All compounds were obtained from Sigma Aldrich.

	**Diet 1**<br>**50%C 6%N**	**Diet 2**<br>**40%C 3%N**	**Diet 3**<br>**30%C 3%N**
Agar	1,23	15,07	38,82
Sucrose	51,43	61,26	37,51
Lysine	1,69	0,84	0,84
Histidine	1,69	0,84	0,84
Leucine	1,69	0,84	0,84
Methionine	1,69	0,84	0,84
Arginine	1,69	0,84	0,84
Valine	1,69	0,84	0,84
Threonine	1,69	0,84	0,84
Isoleucine	1,69	0,84	0,84
Phenylalanine	16,90	8,45	8,45
Tryptophan	16,90	8,45	8,45
Total	100%	100%	100%
